# Supplementary figures and images for: Ferredoxin 1 is essential for embryonic development and lipid homeostasis
Source: eLife. 2024 Jan 22;13:e91656. doi: 10.7554/eLife.91656 (PMC10846857; doi:10.7554/eLife.91656)

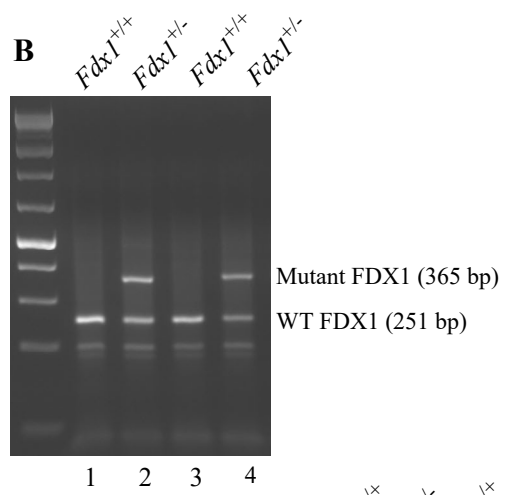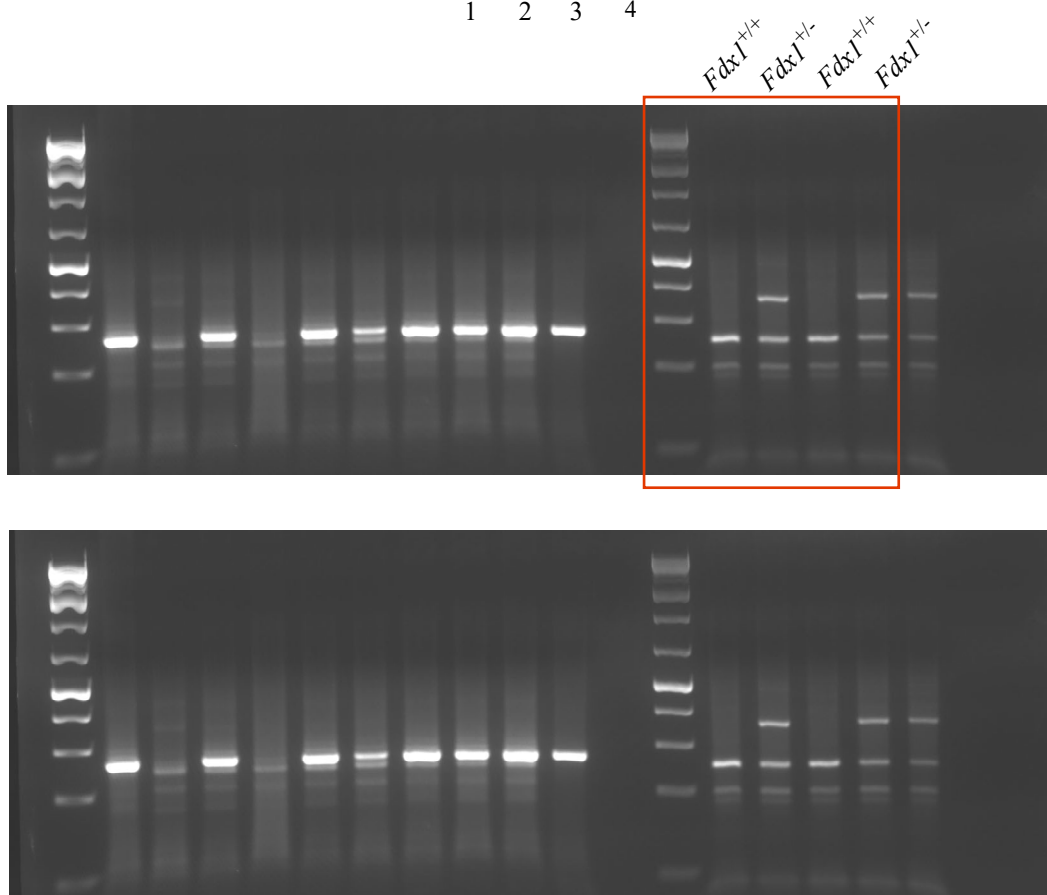

E

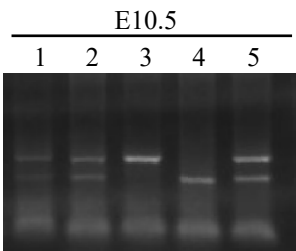

Mutant FDX1  
WT FDX1

E10.5

| 1 | 2 | 3 | 4 | 5 |
|---|---|---|---|---|
|---|---|---|---|---|

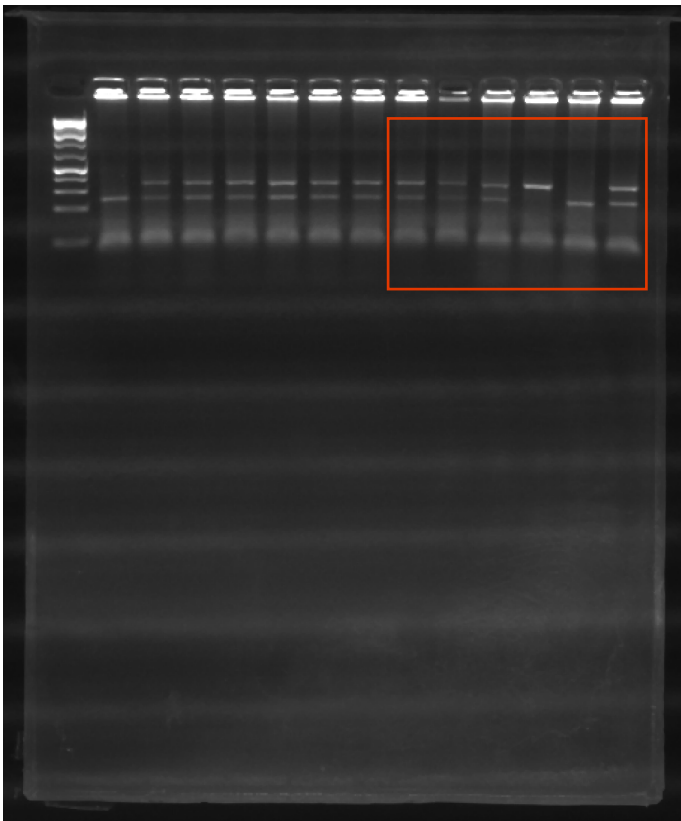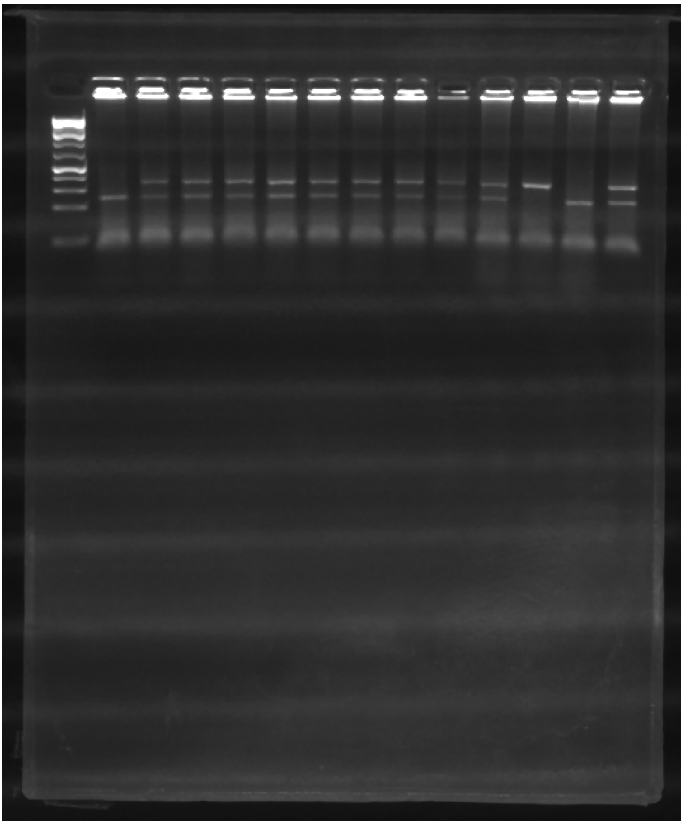

Supplement: Figure 1—source data 1. [file elife-91656-fig1-data1.pdf]

**A**

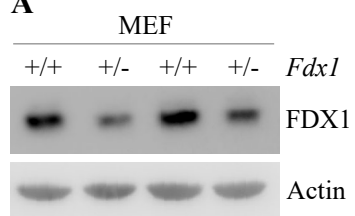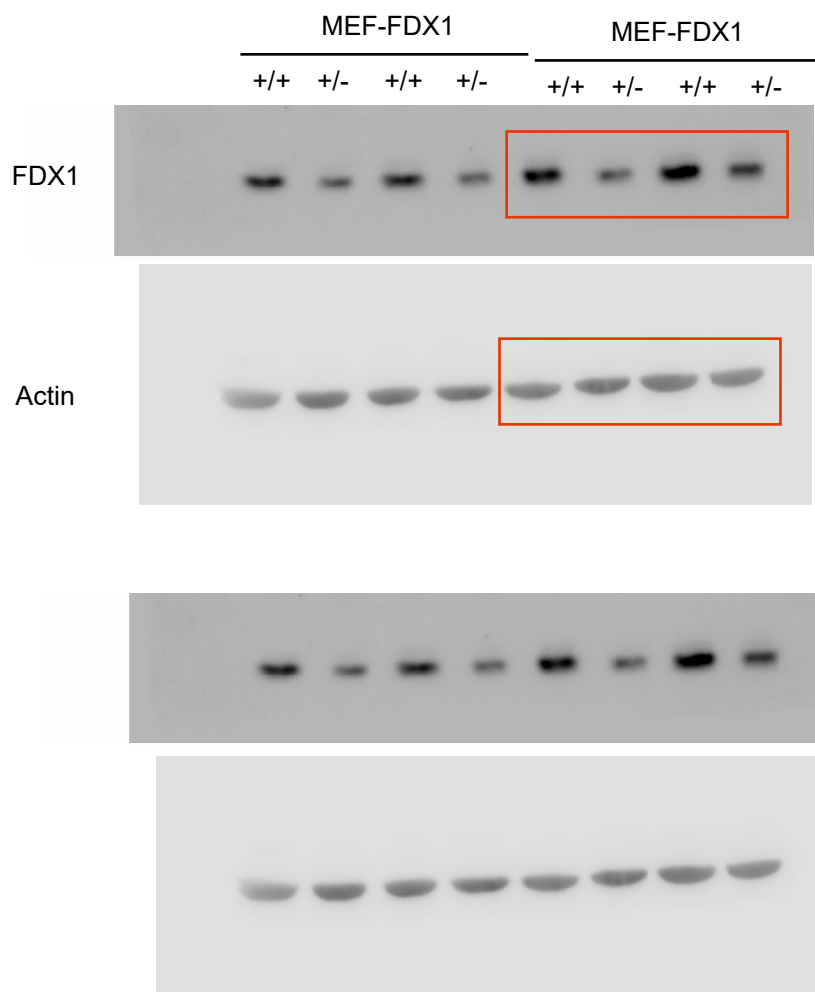

C

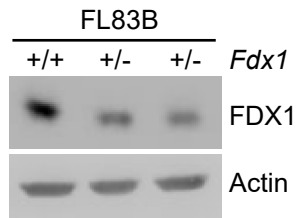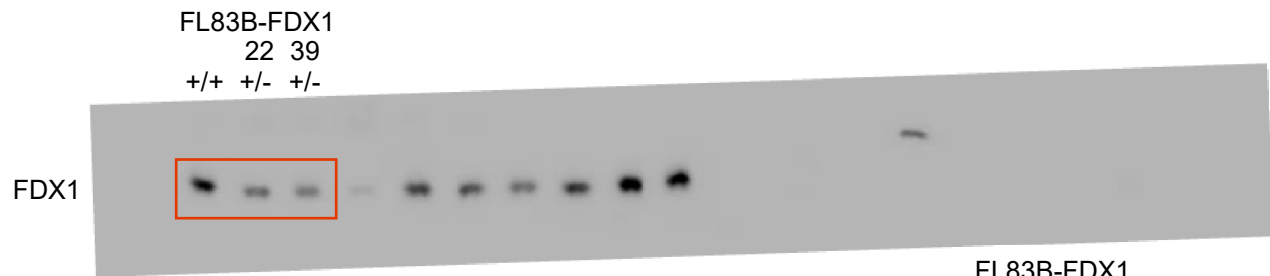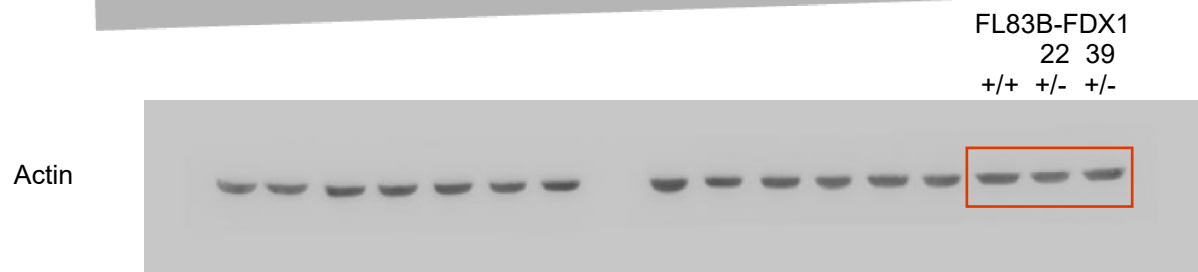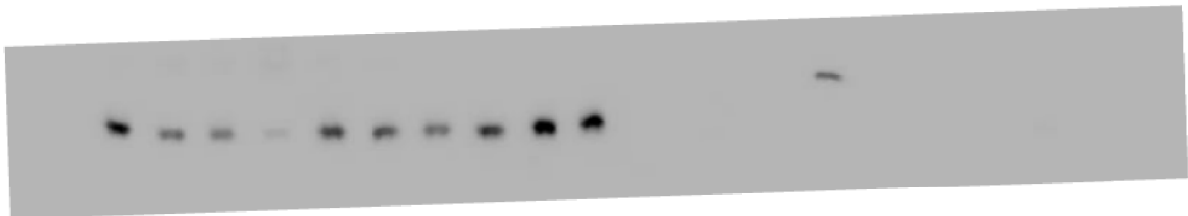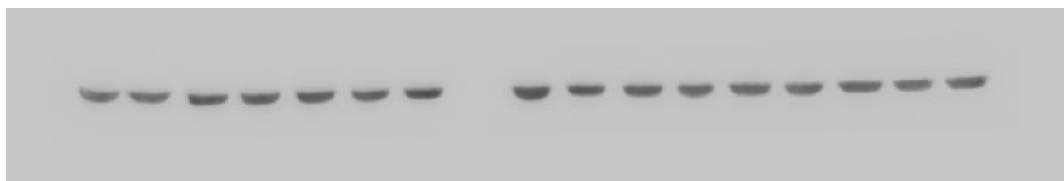

# **E** FL83B

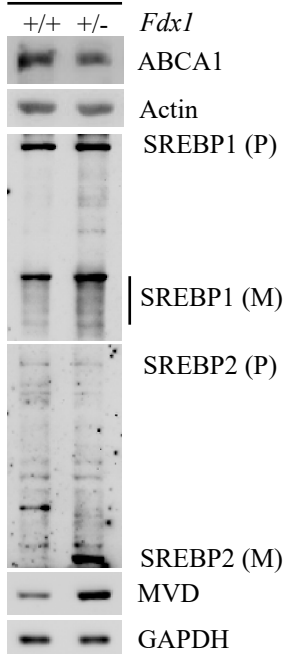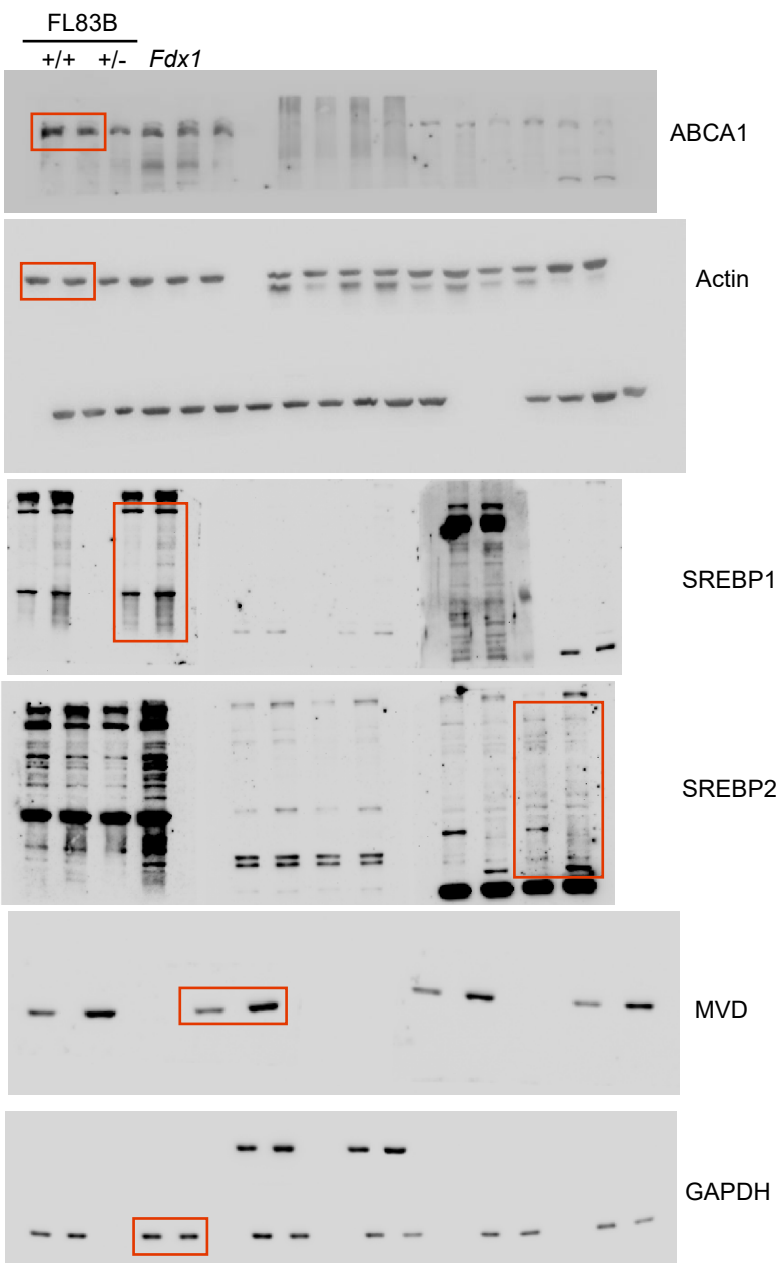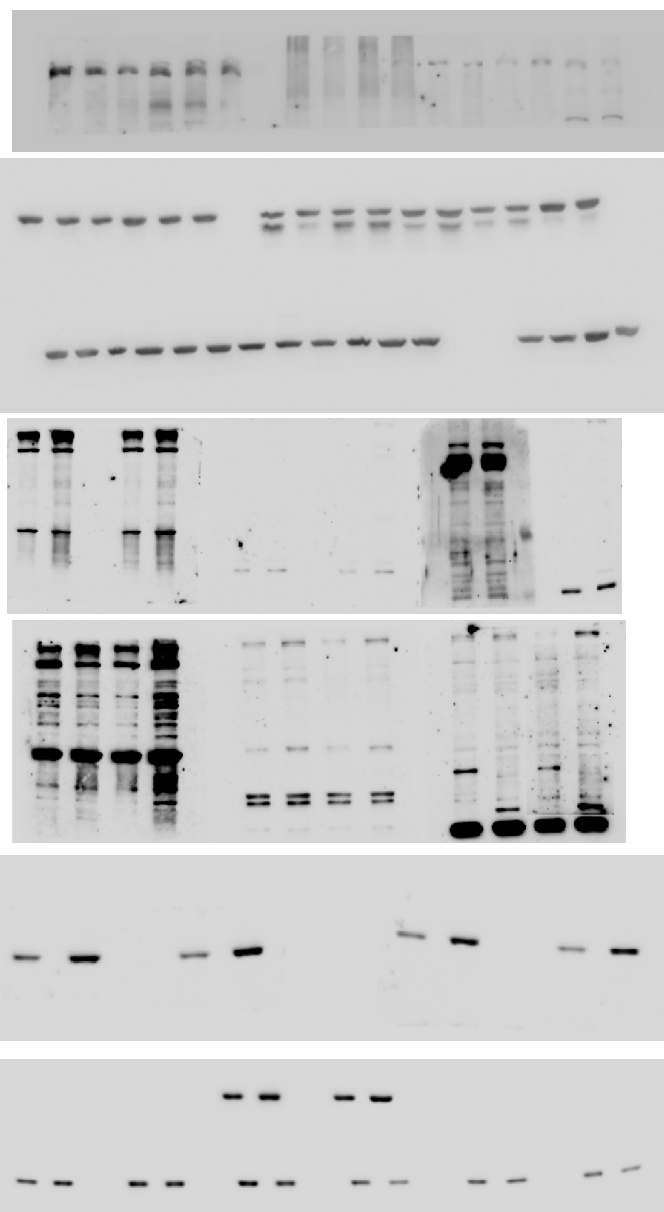

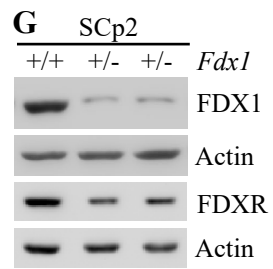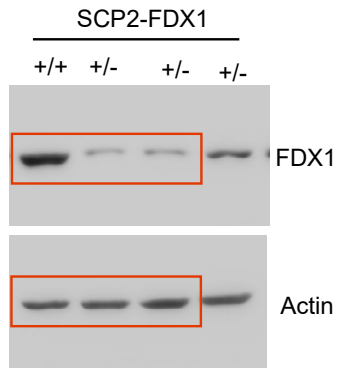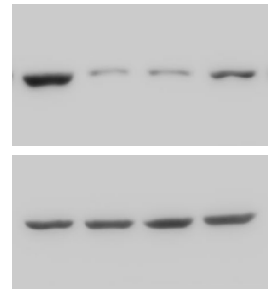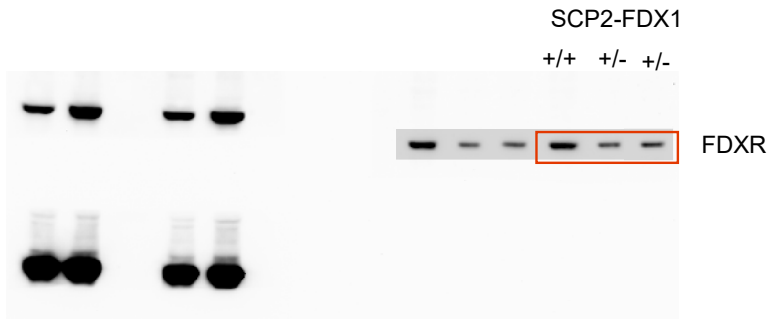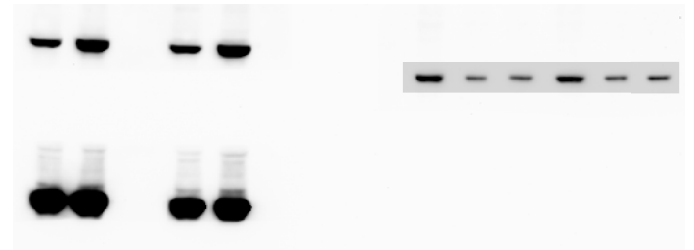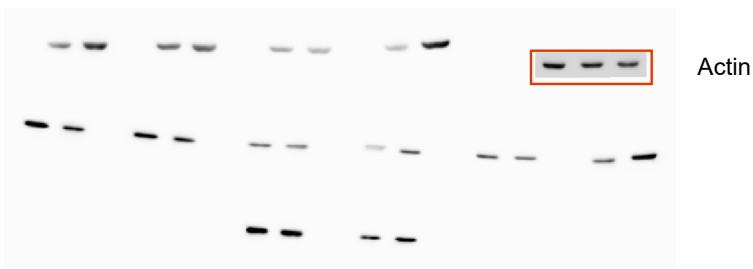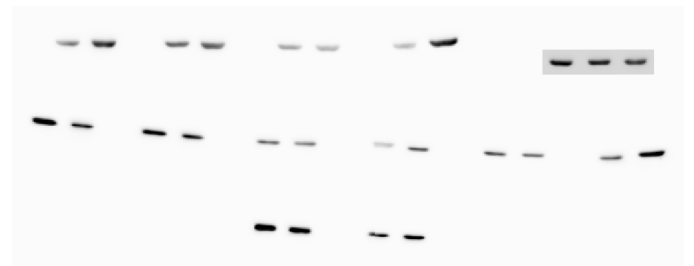

Supplement: Figure 3—source data 1. [file elife-91656-fig3-data1.pdf]

**A**

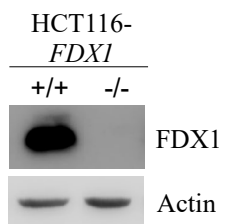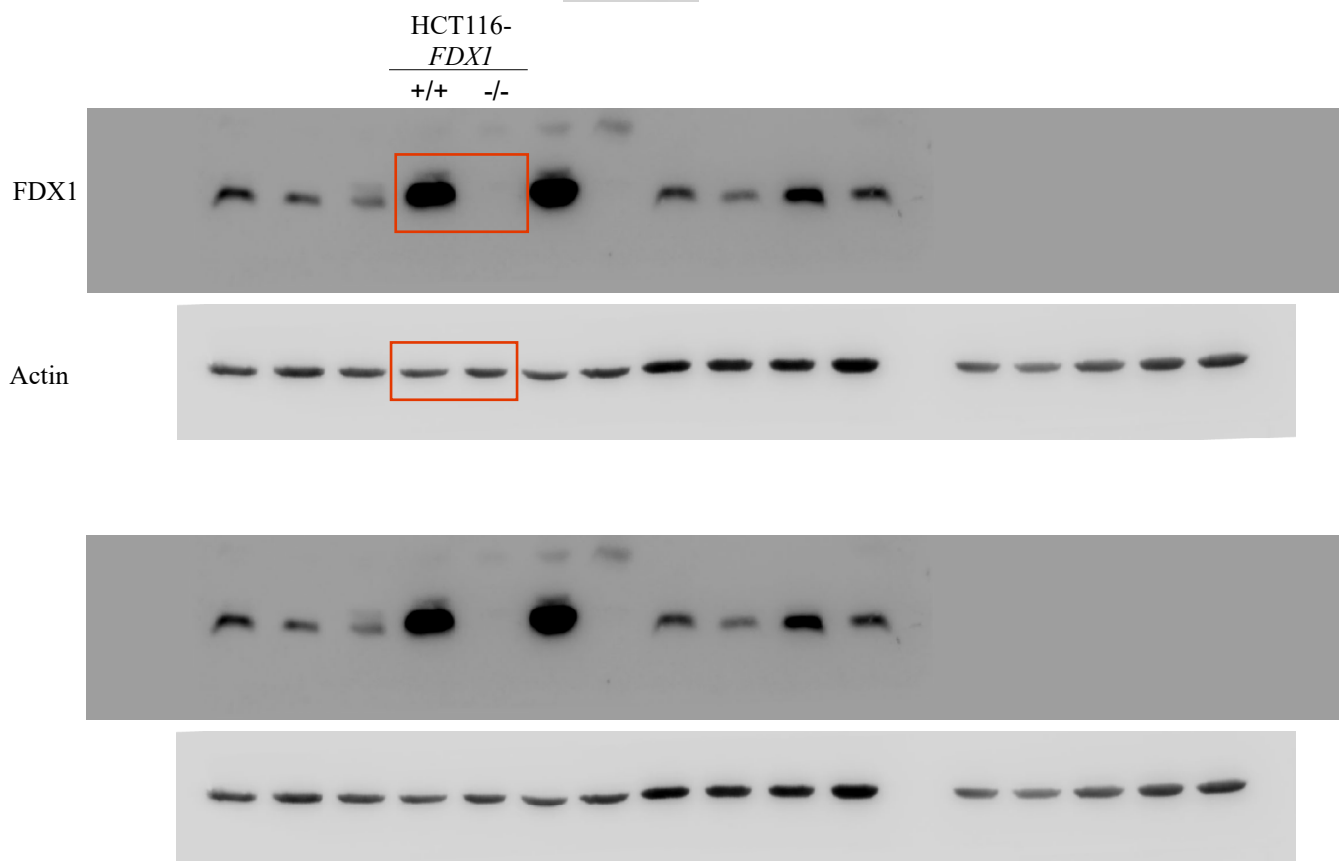

**B**

HCT116-*FDX1*

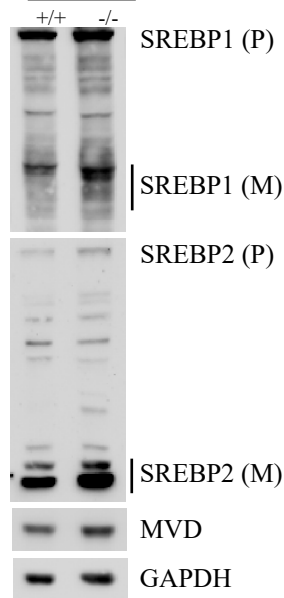

HCT116-*FDX1*

+/+    -/-

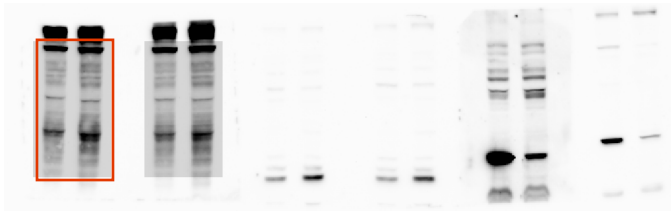

HCT116-*FDX1*

+/+    -/-

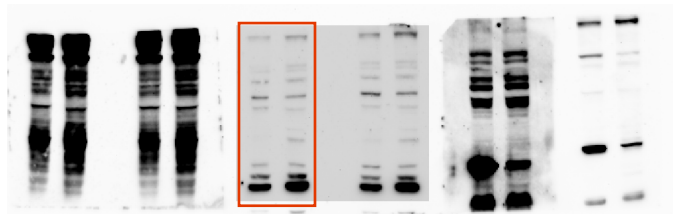

SREBP2

MVD

GAPDH

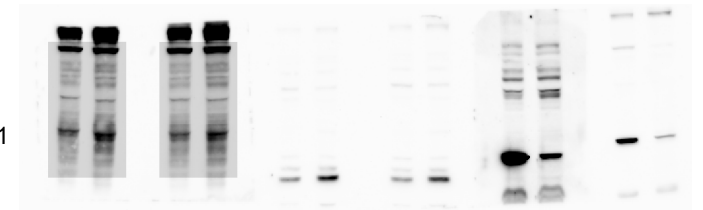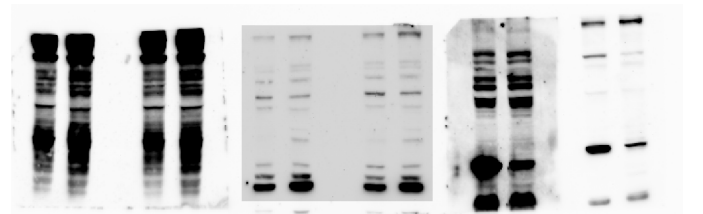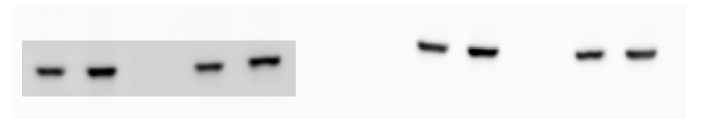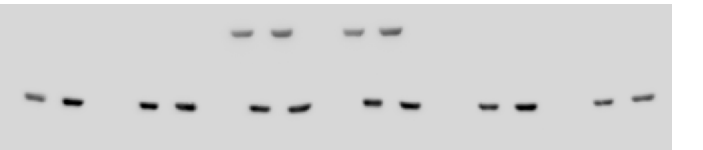

Supplement: Figure 4—source data 1. [file elife-91656-fig4-data1.pdf]

Supplementary Figure S1

A

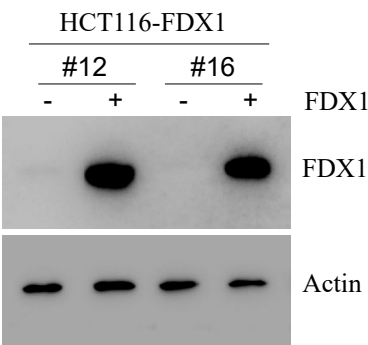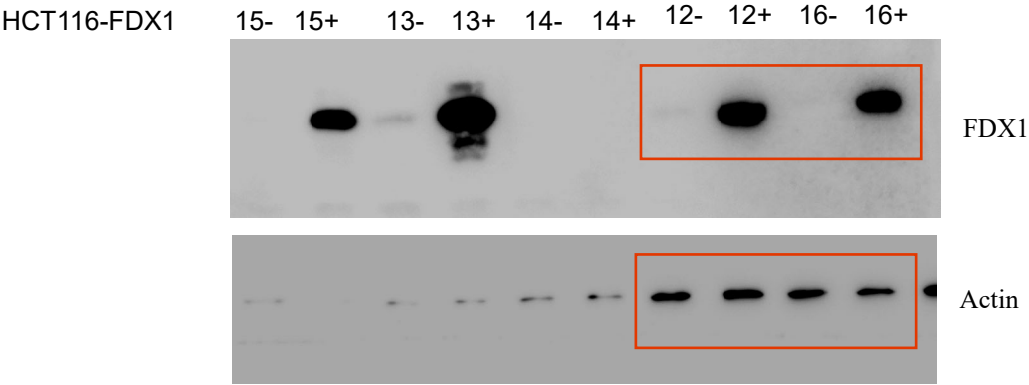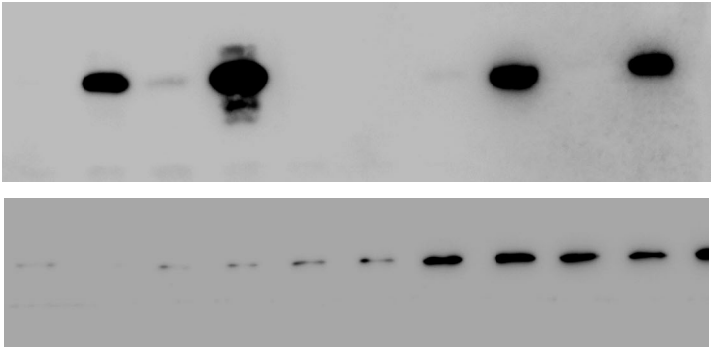

Supplement: Figure 4—figure supplement 1—source data 1. [file elife-91656-fig4-figsupp1-data1.pdf]
