# Supplementary material for: Ferredoxin 1 is essential for embryonic development and lipid homeostasis
Source: eLife. 2024 Jan 22;13:e91656. doi: 10.7554/eLife.91656 (PMC10846857; doi:10.7554/eLife.91656)
Supplement: Figure 2—source data 1. [file elife-91656-fig2-data1.docx]

**Kaplan-Meier Survival Analysis: Log-Rank** Wednesday, July 05, 2023, 12:12:38 PM

**Data source:** Data 1 in Notebook1

**Event labels Censor labels**

1

**Time unit:** None

**Group:** WT

**Event Time No. of Events No. at Risk Probability Std. Error**

83.000 1 56 0.982 0.0177

85.000 1 55 0.964 0.0248

86.000 2 54 0.929 0.0344

90.000 2 52 0.893 0.0413

96.000 1 50 0.875 0.0442

99.000 1 49 0.857 0.0468

100.000 1 48 0.839 0.0491

101.000 1 47 0.821 0.0512

102.000 1 46 0.804 0.0531

103.000 1 45 0.786 0.0548

104.000 1 44 0.768 0.0564

105.000 1 43 0.750 0.0579

106.000 1 42 0.732 0.0592

107.000 1 41 0.714 0.0604

109.000 3 40 0.661 0.0633

111.000 3 37 0.607 0.0653

113.000 2 34 0.571 0.0661

115.000 1 32 0.554 0.0664

116.000 1 31 0.536 0.0666

117.000 5 30 0.446 0.0664

119.000 1 25 0.429 0.0661

120.000 2 24 0.393 0.0653

121.000 2 22 0.357 0.0640

122.000 2 20 0.321 0.0624

124.000 1 18 0.304 0.0614

126.000 1 17 0.286 0.0604

127.000 4 16 0.214 0.0548

128.000 1 12 0.196 0.0531

129.000 3 11 0.143 0.0468

130.000 1 8 0.125 0.0442

132.000 1 7 0.107 0.0413

133.000 1 6 0.0893 0.0381

134.000 2 5 0.0536 0.0301

140.000 1 3 0.0357 0.0248

143.000 1 2 0.0179 0.0177

144.000 1 1 0.000 0.000

Number of Cases 56

Missing Values 0

Events 56

Censored 0

% Censored 0

**Survival Time Std. Error 95% Conf. Lower 95% Conf. Upper**

Mean 115.232 2.001 111.310 119.155

Percentiles:

25 127.000 2.047 122.988 131.012

50 (Median) 117.000 1.240 114.570 119.430

75 106.000 2.900 100.317 111.683

**Group:** Fdxr+/-

**Event Time No. of Events No. at Risk Probability Std. Error**

39.000 1 31 0.968 0.0317

60.000 1 30 0.935 0.0441

68.000 1 29 0.903 0.0531

77.000 1 28 0.871 0.0602

86.000 1 27 0.839 0.0661

90.000 1 26 0.806 0.0710

92.000 1 25 0.774 0.0751

97.000 1 24 0.742 0.0786

98.000 1 23 0.710 0.0815

100.000 4 22 0.581 0.0886

102.000 3 18 0.484 0.0898

104.000 1 15 0.452 0.0894

106.000 1 14 0.419 0.0886

108.000 1 13 0.387 0.0875

112.000 1 12 0.355 0.0859

113.000 1 11 0.323 0.0840

114.000 2 10 0.258 0.0786

115.000 3 8 0.161 0.0661

116.000 2 5 0.0968 0.0531

119.000 1 3 0.0645 0.0441

123.000 1 2 0.0323 0.0317

125.000 1 1 0.000 0.000

Number of Cases 31

Missing Values 0

Events 31

Censored 0

% Censored 0

**Survival Time Std. Error 95% Conf. Lower 95% Conf. Upper**

Mean 100.903 3.401 94.238 107.569

Percentiles:

25 115.000 0.819 113.395 116.605

50 (Median) 102.000 3.339 95.456 108.544

75 97.000 3.480 90.179 103.821

**Group:** Fdx1+/-

**Event Time No. of Events No. at Risk Probability Std. Error**

7.700 1 26 0.962 0.0377

16.300 1 25 0.923 0.0523

18.900 1 24 0.885 0.0627

47.700 1 23 0.846 0.0708

51.000 1 22 0.808 0.0773

59.900 2 21 0.731 0.0870

63.700 1 19 0.692 0.0905

65.600 1 18 0.654 0.0933

69.900 1 17 0.615 0.0954

71.100 1 16 0.577 0.0969

72.600 1 15 0.538 0.0978

73.400 1 14 0.500 0.0981

75.100 1 13 0.462 0.0978

77.600 2 12 0.385 0.0954

78.700 2 10 0.308 0.0905

88.600 1 8 0.269 0.0870

92.000 1 7 0.231 0.0826

93.900 1 6 0.192 0.0773

104.000 3 5 0.0769 0.0523

109.700 1 2 0.0385 0.0377

119.000 1 1 0.000 0.000

Number of Cases 26

Missing Values 0

Events 26

Censored 0

% Censored 0

**Survival Time Std. Error 95% Conf. Lower 95% Conf. Upper**

Mean 72.331 5.479 61.591 83.070

Percentiles:

25 92.000 10.885 70.666 113.334

50 (Median) 73.400 3.314 66.904 79.896

75 59.900 9.575 41.134 78.666

**Data Summary:**

**Group Total Missing Events Censored Percent Censored Median Time**

WT 56 0 56 0 0 117.000

Fdxr+/- 31 0 31 0 0 102.000

Fdx1+/- 26 0 26 0 0 73.400

Overall 113 0 113 0 0

**Log-Rank Test:**

**Statistic DF P Value**

71.959 2 <0.001

The log rank statistic for the survival curves is greater that would be expected by chance; there is a statistically significant difference between survival curves (P = <0.001).

To isolate the group or groups that differ from the others use a multiple comparison procedure.

**Multiple Comparisons:**

All Pairwise Multiple Comparison Procedures (Holm-Sidak method):

Overall significance level = 0.05

**Comparisons Statistic P Value Significant?**

WT vs. Fdx1+/- 62.482 7.994E-015 Yes

WT vs. Fdxr+/- 19.406 0.0000211 Yes

Fdxr+/- vs. Fdx1+/- 15.098 0.000102 Yes
